# Towards an Integrative Model of Math Cognition: Interactions between Working Memory and Emotions in Explaining Children’s Math Performance

**DOI:** 10.3390/jintelligence11070136

**Published:** 2023-07-07

**Authors:** Sanne H. G. Van der Ven, Emilie J. Prast, Eva Van de Weijer-Bergsma

**Affiliations:** 1 Behavioural Science Institute, Radboud University Nijmegen, Thomas van Aquinostraat 4, 6525 GD Nijmegen, The Netherlands; 2 Faculty of Social and Behavioral Sciences, Institute of Education and Child Studies, Leiden University, Wassenaarseweg 52, 2333 AK Leiden, The Netherlands; e.j.prast@fsw.leidenuniv.nl; 3 Faculty of Social and Behavioral Sciences, Department Education and Pedagogy, Utrecht University, Heidelberglaan 1, 3584 CS Utrecht, The Netherlands; e.vandeweijer@uu.nl

**Keywords:** mathematics, arithmetic, working memory, anxiety, enjoyment, primary school children

## Abstract

Individual variation in mathematical skills can be ascribed to differences in cognitive ability, but also to students’ emotional experiences of mathematics, such as enjoyment and anxiety. The current study investigated how the interplay of working memory with math anxiety and enjoyment explains mathematical performance in primary school students. We also explored whether these relations differed with the type of math test and students’ age. Using mixed effect models, we reanalyzed data from 4471 Dutch primary school students (grades 2–6) who had completed two computerized working memory tasks, had filled out a questionnaire on math emotions, and had completed two math tests: story problems and speeded arithmetic. Findings showed that working memory, anxiety, and enjoyment were linear (but not curvilinear) predictors of performance on both tests, while some relations were stronger for the math (story)-problem-solving test. Higher math anxiety negatively impacted performance more strongly for students with stronger working memory skills, but only on the arithmetic test. No interaction between working memory and enjoyment was found. The relation between math anxiety and math performance increased with grade level, but no other age-related changes were found. Interpretations and recommendations focus on situated views on learning and emotion.

## 1. Introduction

Mathematical abilities are crucial for achieving career success. However, there is already considerable individual variation in math ability at an early age. This variation can be partially ascribed to differences in cognitive ability, in particular working memory (Namkung et al. 2019; Peng et al. 2016; Raghubar et al. 2010). However, children also differ in their emotional experience of mathematics, for example, the degree of enjoyment and anxiety they experience (Dowker et al. 2016; Putwain et al. 2018; Raccanello et al. 2019). In the present study, we investigated how the interplay of both cognitive and affective factors explains mathematical performance in children in primary school. While the importance of each of these factors has been demonstrated previously, the present study strives towards a more integrative model to explain how enjoyment and anxiety interact with working memory in predicting students’ mathematics performance across the primary school years.

### 1.1. Working Memory and Mathematics

Mathematical problem solving requires working memory (WM) resources in different ways. Examples are the retrieval of numerical information from long-term memory, carrying out numerical manipulations, remembering answers to partial problems, and creating a problem representation and keeping track of the different steps in multistep problem solving. Many empirical studies and meta-analyses have confirmed that individual differences in WM ability are an important predictor of variation in mathematics performance: higher WM ability is associated with stronger math skills (Friso-Van den Bos et al. 2013; Geary et al. 2017; Holmes and Adams 2006; Lee and Bull 2016; Passolunghi and Pazzaglia 2004; Peng et al. 2016; Swanson and Kim 2007; Van der Ven et al. 2012, 2013). Although, traditionally, it was assumed that WM unidirectionally affects later math performance, more recently, a more reciprocal and dynamic relation is assumed: WM ability can improve as a result of children using their WM in solving math problems (Kahl et al. 2022; Peng and Kievit 2020).

The level of involvement of WM depends on the type of math activity (Allen et al. 2019; Friso-Van den Bos et al. 2013). WM is especially important in complex math problems that require difficult, multistep procedures, rather than the retrieval of answers to memorized problems. Story problems that require math problem solving—including the construction of a mental model of the situation and the problem to be solved—thus draw more heavily on WM compared to performing numerical calculations (arithmetic), which are more often (partially) memorized.

### 1.2. Emotions and Mathematics

Mathematical problem solving is also impacted by the emotions that children experience in relation to learning activities and achievement. According to the control–value theory (CVT; Pekrun 2006), achievement emotions are shaped by cognitive appraisals, specifically the extent to which learners experience control over achievement activities and their outcomes, and the value they ascribe to those activities and outcomes (Pekrun 2006; Pekrun and Perry 2014). It is theorized that achievement emotions influence achievement indirectly through motivation and information processing.

There is a range of achievement emotions, including positive emotions (enjoyment, and pride) and negative emotions (anxiety, shame, boredom, anger, and hopelessness). Positive emotions are typically positively related to mathematics performance, whereas negative emotions are negatively related to mathematics performance (Pekrun et al. 2017). While positive and negative emotions have independent effects on performance, the pattern of effects is similar for the various emotions within each category (positive or negative affect; Pekrun et al. 2017). In the current study, we focus on one positive emotion (enjoyment) and one negative emotion (anxiety) because we suspect that these specific emotions interact with working memory, as we will argue in the following sections.

#### 1.2.1. Anxiety and Mathematics

Children with math anxiety experience feelings of tension, worry, and fear in situations involving math content or activities (Wang et al. 2015). Several recent reviews and meta-analyses invariably concluded that math anxiety increases with age in children, and that there is an overall moderately sized negative relation between math anxiety and math performance: higher levels of math anxiety are related to lower performance on math achievement tests (Barroso et al. 2021; Dowker et al. 2016; Namkung et al. 2019; Zhang et al. 2019).

Math anxiety and performance have a reciprocal relation: poor performance can trigger math anxiety, and anxiety can impair subsequent performance (Carey et al. 2016). Some longitudinal studies have shown that lower math performance was a predictor of higher math anxiety one year later (e.g., Gunderson et al. 2018; Zhang et al. 2023), while the reverse relation seems also present but weaker: anxiety is less predictive of later math performance. There are multiple potential mechanisms for the effect of anxiety on performance. First, anxiety leads students to avoid taking mathematics classes when they can (Hembree 1990). However, a second mechanism is that anxiety directly disturbs performance through the depletion of cognitive resources (Finell et al. 2022). Anxiety is often accompanied by intrusive thoughts and worry, creating a dual-task situation in which fewer attentional and WM resources are available for math performance (Ashcraft and Krause 2007; Beilock 2008; Eysenck et al. 2007; Namkung et al. 2019). Experimental studies have shown that an anxiety-inducing intervention directly reduces math performance (Beilock 2008; Beilock et al. 2004), a phenomenon called “choking under pressure” (Beilock 2008; Beilock and Carr 2001). Moreover, the relation between anxiety and performance is stronger for high-stakes math tests than for research-only (and thus low-stakes) math tests (Namkung et al. 2019), and stronger in a timed than in an untimed task (Faust 1996), suggesting that certain tests induce stronger choking effects. Research also indicates that the relation between math anxiety and math performance depends on the difficulty of the math activity, presumably since tasks requiring more complex problem solving tax cognitive resources more strongly compared to simple tasks, such as simple arithmetic problems (Ching 2017; Namkung et al. 2019).

Research shows that math anxiety is especially detrimental for students with a high WM ability (Beilock and Carr 2005). While high WM in general leads to better math performance, anxiety prevents especially these higher-ability students from reaching their full potential. In stress-free circumstances, those with a high WM ability can apply complex strategies that pose a large load on WM. However, in stressful circumstances, WM is compromised and these complex strategies no longer work (Ramirez et al. 2016). Consequently, math problem solving in persons with high WM ability suffers more from anxiety and stress than persons with low WM, who generally do not apply these complex strategies in stress-free circumstances either (Beilock and Carr 2005). This finding of persons with high WM suffering disproportionally from anxiety is supported by both correlational, often longitudinal, studies (Ching 2017; Ramirez et al. 2016; Vukovic et al. 2013) and experimental studies, in which the performance of the same persons is compared in an anxiety-provoking condition and a control condition (Beilock and Carr 2005). In both types of studies, stress and anxiety are found to exert a larger effect in those with better WM ability.

Less is known about at which levels anxiety negatively affects performance. Similar to positive stress, intermediate levels of arousal as a result of anxiety may actually enhance performance (Rudland et al. 2020). An inversely U-shaped curve, also called the Yerkes–Dodson law (Yerkes and Dodson 1908), is often used to describe stress and performance. According to this model, performance is optimal when arousal is intermediate, and performance is lower under both low and high levels of arousal. Especially for high-WM students, some degree of math anxiety may be better than no anxiety, but a high level of math anxiety is most detrimental.

#### 1.2.2. Enjoyment and Mathematics

Math is certainly not always a negative, anxiety-provoking experience. In recent years, there has been a surge in studies investigating positive emotions in mathematics. Invariably, these studies have shown a positive relation between mathematics enjoyment and mathematics performance (Frenzel et al. 2007; Putwain et al. 2018; Raccanello et al. 2019; Van der Beek et al. 2017; Villavicencio and Bernardo 2015). Enjoyment is negatively related to anxiety, but only moderately (Raccanello et al. 2019; Van der Beek et al. 2017; Villavicencio and Bernardo 2015), warranting the treatment of both emotions as separate constructs.

Similar to math anxiety, current insights suggest that the relation between enjoyment and math performance is reciprocal (Pekrun et al. 2017; Pinxten et al. 2014; Putwain et al. 2018). On the one hand, high achievement predicts later enjoyment (Goetz et al. 2008; Pekrun et al. 2017; Pinxten et al. 2014; Putwain et al. 2018). In line with a control–value perspective (Pekrun et al. 2017), when students show better performance, they experience more feelings of competence and control over mathematical situations, which in turn promotes their enjoyment (Goetz et al. 2008; Van der Beek et al. 2017). On the other hand, higher enjoyment of mathematics predicts subsequent higher achievement (Ahmed et al. 2013; Pekrun et al. 2017; Pinxten et al. 2014; Putwain et al. 2018). Enjoyment is assumed to affect achievement through positive effects on learning processes and behaviors (Pekrun 2006), including increased effort expenditure (Pinxten et al. 2014) and more use of deep and metacognitive learning strategies (Ahmed et al. 2013). To our knowledge, there are no studies examining if math enjoyment predicts math performance differently depending on the type of math test. However, a study by Sundre and Kitsantas (2004) on the effects of motivation (a construct related to enjoyment) suggests that motivational aspects are more relevant in low-stakes tests compared to high-stakes tests. The authors theorize that a high-stakes test triggers effort in all students, while effort on low-stakes tests depends more strongly on (intrinsic) motivation. More generally, positive emotions, including enjoyment, are theorized to free up cognitive resources (“broaden-and-build”) and, therefore, enhance flexible information processing (Fredrickson 2001; Isen 2004; Pekrun 2006). In line with this theorized mechanism, empirical studies have showed that a positive mood induction increased performance on a WM task (Storbeck and Maswood 2015; Yang et al. 2013), that trait positive affect was positively related to WM ability (Figueira et al. 2018), and that natural fluctuations in positive mood covaried with WM performance within individuals (Brose et al. 2014).

Given this association between enjoyment and information processing, it is relevant to investigate whether, analogous to anxiety, enjoyment and WM ability interact in their impact on achievement. As far as we know, this question has not yet been addressed in empirical research. If enjoyment frees up cognitive resources, children with limited WM ability might profit from enjoyment to a different extent, although the direction of effects is not clear a priori. On the one hand, children with limited WM ability might profit more from enjoyment, since this positive affective state might enable them to use their limited WM capacity more effectively, thus enabling them to perform math tasks that would otherwise impose too much cognitive load. Alternatively, enjoyment might be especially beneficial for children with high WM ability (as seems to be the case for anxiety), because of their tendency to use WM intensive solution strategies, for which the potential gain from freeing up cognitive resources is larger.

Finally, also for enjoyment, it is unknown whether its effect on performance is linear or curvilinear. There might be an optimum level, after which increased enjoyment leads to distraction and lower math performance, because irrelevant elements of the activity might enter WM. This question has received only little attention thus far. One study tested both the linear and quadratic effects of positive affect on WM performance, and only the linear effect was significant (Brose et al. 2014). However, an inverse U-shaped curve is often found in the relation between WM performance and dopamine (Cools and D’Esposito 2011), a neurotransmitter associated with motivational and attentional processes (Westbrook et al. 2020). To our knowledge, curvilinear relations with math performance have not been investigated yet.

### 1.3. The Effect of Age

Age-related differences in the patterns described above may also be relevant since children’s emotions may evolve as a result of their repeated experiences with math learning while progressing through primary school. This may lead to a cascade of interrelations strengthening over time (Cargnelutti et al. 2017). Although research shows that math anxiety increases as children grow older, it remains unclear if its predictive value for math performance also becomes stronger. While the meta-analysis by Barroso et al. (2021) shows a stronger relation between math anxiety and performance in higher grades, the meta-analysis by Namkung et al. (2019) does not reveal such an age-related difference. This may be due to the relatively low number of studies in young children, but might also be explained by smaller variance in math anxiety in younger children, possibly because young children’s self-awareness and metacognitive skills are still underdeveloped (Li et al. 2021).

The rare studies on age-related changes in enjoyment show that although children experience less math enjoyment as they grow older (Lichtenfeld et al. 2022; Raccanello et al. 2019; Vierhaus et al. 2016), the relation between math enjoyment and achievement remains similar from grade 2 to 4 (Lichtenfeld et al. 2022).

Research on age-related changes in WM reveals that it remains an important predictor of mathematics performance throughout childhood (Geary et al. 2017; Lee and Bull 2016) and the strength of its effect is relatively stable over age (Cragg et al. 2017; Lee and Bull 2016).

However, the association between math anxiety and WM seems to become stronger as children grow older (Finell et al. 2022). Children who have experienced more failure in math (possibly due to lower WM ability) may develop stronger math anxiety, which could hamper their subsequent math learning even more. Additionally, as math problems become more complex in higher grade levels, WM resources may be taxed more, increasing the potential detrimental effects of math anxiety.

### 1.4. The Present Study

In sum, while the interplay between anxiety and WM in math learning has been well established, less is known about the role of enjoyment. Moreover, the impact of emotions on cognitive resources may depend on how strongly these emotions are experienced and on age. The present study aims to create a broader framework on how varying levels of negative and positive math-related emotions interact with WM to affect math performance, and how these relations change with age. We will examine these relations using two types of math tasks: a speeded but low-stakes arithmetic task and a high-stakes standardized mathematics achievement test that involves more complex word problems.

First, we examine the main effects of WM, anxiety, enjoyment, and WM on mathematics achievement. We expect that stronger WM ability predicts higher math performance. We expect a negative main effect of math anxiety on math performance that may be curvilinear (concave, i.e., a negative quadratic effect) since a slight-to-moderate degree of anxiety may increase alertness and enhance performance. Enjoyment is expected to have a positive main effect on math performance since it enhances attentional quality during math class. This effect might be curvilinear too, but we do not have a hypothesis on the exact shape of the curve.

Second, we investigate the interaction effects between WM and the emotions of anxiety and enjoyment. We expect that the negative effect of anxiety on math achievement is stronger in children with higher WM ability, as shown in the previous literature (Ramirez et al. 2013). Furthermore, there may be quadratic moderation effects of WM and anxiety: the stronger (negative) effect of anxiety in children with higher WM may only become visible at higher levels of anxiety, as it will hamper their ability to perform complex calculations. On the other hand, slight or moderate anxiety may have positive effects in children with higher WM as manageable stress can increase alertness and performance. We also investigate the interaction between WM and enjoyment, but we do not have a hypothesis for its direction. On the one hand, the positive effect of enjoyment on math achievement may be stronger for children with low WM ability, because for them, it is especially important to use their limited resources effectively. On the other hand, children with high WM abilities may use complex strategies that work optimally in a positive, broaden-and-build mood. Following this theory, the positive effect of enjoyment will be stronger for children with high WM. Again, we will explore possible quadratic interaction effects between WM and enjoyment.

Third, we investigate if these relations differ with age (i.e., grade level). We expect the relations to become stronger over time, because older children have had more time for accumulating the effects of both anxiety and enjoyment.

Finally, we explore whether the type of math task matters: does the strength of the abovementioned effects differ between the speeded arithmetic task and the math-problem-solving test? WM is assumed to be more strongly related to the math-problem-solving task than the arithmetic task, as the latter requires less complex strategies. Our expectations regarding emotions are more exploratory: although previous research suggests that math anxiety may be higher in the math-problem-solving task, requiring more complex reasoning (Ching 2017; Namkung et al. 2019), the speeded nature of the arithmetic task may trigger higher stress despite involving more simple calculations. Hypotheses about how emotions and WM interact differentially between the two types of test can also be formulated in two directions: For example, if math problem solving places higher demands on WM than the arithmetic task, math anxiety may hamper WM more strongly during the math-problem-solving test as children need all their cognitive resources to solve them. Moreover, as the math-problem-solving task is part of the regular testing policy, students may experience this task as high-stakes. On the other hand, if the (research-only) speeded arithmetic task triggers higher levels of math anxiety, this may hamper performance on this test more, even though it poses a lower demand on WM.

## 2. Materials and Methods

The present study is a reanalysis of an existing data set. Data were collected in the context of the large-scale project GROW (Prast et al. 2018a) with a longitudinal design. Prior to the reanalysis of the data, the analysis protocol was registered in the open science framework (https://osf.io/wx7dt).

### 2.1. Participants

Thirty-two schools spread across the Netherlands volunteered to participate in this project about differentiation in primary mathematics education. Because of the large scale of the study, a passive consent procedure was used, in which parents received written information about the study. Parents informed the teacher of their child in case they did not allow their child to participate. This was in line with consent regulation at that time and approved by the ethics committee of the Social and Behavioral Sciences Faculty, Utrecht University (Approval code: 22-0070). The selected sample consisted of 4471 children (50.6 % male) nested in 184 classes (mean class size = 24 students) from grade 2 to 6. The mean age at the beginning of the study was 9 years and 5 months (SD = 1 year and 6 months).

### 2.2. Materials

#### 2.2.1. Math Emotions

Math anxiety and enjoyment were assessed with subscales from the Mathematics Motivation Questionnaire for Children (MMCQ; Prast et al. 2012, 2018b). This self-report questionnaire was designed to measure several aspects of motivation for mathematics in primary school students. Math anxiety was assessed with 5 items concerning anxious thoughts and feelings during the mathematics lesson, e.g., “During math class, are you afraid of making mistakes?”. For math enjoyment, 5 items were selected from a 7-item subscale that measured task value: e.g., “Do you like math?” and “Do you usually look forward to math class?”. The remaining items measured personal value and utility value and were, therefore, not relevant to the current study. All items were rated on the following four-point scale: 1 = NO! (strongly disagree), 2 = no (disagree), 3 = yes (agree), 4 = YES! (strongly agree). The internal consistency of the scales is good-to-excellent (Cronbach’s α ranges between 0.79 and 0.87 for math anxiety and between 0.89 and 0.93 for enjoyment) in the current sample. The test–retest reliability for the complete MMCQ over a 1-week period was good (r ranges from 0.82 to 0.93 for different scales; Prast et al. 2018b). Negatively worded items were score-reversed, and then a mean for each scale was calculated, ranging from 1 to 4, with a higher score reflecting higher math anxiety/math enjoyment.

#### 2.2.2. Working Memory

Children completed two online computerized WM tasks, the Lion game (Van de Weijer-Bergsma et al. 2015) and the Monkey game (Van de Weijer-Bergsma et al. 2016), suitable for self-administration in the classroom. The Lion game is a visual–spatial complex span task in which children remember the location of colored lions. In each trial, eight lions of different colors (red, blue, green, yellow, and purple) are presented sequentially for 2000ms at different locations in a 4 × 4 matrix containing 16 bushes. Children have to remember the last location where a lion of a certain color (e.g., red) has appeared. After the trial, children click on the correct location. The WM load of the task increases from level 1 to level 5 by increasing the number of colors—and thus the number of locations—children have to remember and update.

The Monkey game is a backward verbal span task, in which children hear spoken one-syllable words (Van de Weijer-Bergsma et al. 2016). Children have to remember the words and recall them in reversed order by clicking on the words presented visually in a 3 × 3 matrix. The WM load of the task increases from level 1 to level 5 by increasing the number of words children have to remember (ranging from two to six words).

Both tasks consist of 20 items in total (4 items per level). No cut-off rules are applied. We scored the proportion of items recalled correctly. The Lion and Monkey games have excellent (Cronbach’s α between 0.86 and 0.90 for different ages) and good internal consistency (Cronbach’s α between 0.78 and 0.89 for different ages), respectively, and good concurrent and predictive validity (cf. Van de Weijer-Bergsma et al. 2015, 2016). The test–retest reliability (ρ = 0.71) for the Lion game is satisfactory. The Monkey game shows substantial stability over a period of two years (SE = 0.52, *p* < .001, after controlling for age SE = 0.41, *p* < .001).

### 2.3. Mathematics

#### 2.3.1. Math Problem Solving

The criterion-based Cito Mathematics Tests (Cito Math) are tests used to monitor the progress of Dutch primary school children (Janssen et al. 2005). These tests primarily consist of contextual math problems. There are two different versions for each grade: one to be administered at the middle of the school year and one at the end of the school year. In each test, five main domains are covered: (a) numbers and number relations, (b) addition and subtraction, (c) multiplication and division, (d) complex math applications, often involving multiple mathematical manipulations, and (e) measuring (e.g., weight and length). From grade 2 to grade 6, several domains are integrated in the math curricula successively: (f) estimation, (g) time, (h) money, (i) proportions, (j) division, and (k) percentages. The difficulty level of the items for each domain increases with grade level. Raw test scores are converted to competence scores that increase throughout primary school, enabling the comparison of results of different tests on the same scale (Janssen et al. 2005). Competence scores can vary between 0 (lowest in grade 1) and 169 (highest in grade 6). The reliability for the math tests in different grades ranges from 0.91 to 0.97 (Janssen et al. 2010).

#### 2.3.2. Speeded Arithmetic

The Arithmetic Tempo Test (De Vos 1992) is a standardized speeded paper-and-pencil arithmetic test to measure math fluency. The test consists of five sets of 40 addition (+), subtraction (−), multiplication (×), and division (÷) problems, and a mixture of the four domains. For each set, children have 1 min to solve as many problems as possible. All problems consist of two-operand equations with an outcome smaller than 100 and both operands ranging between 0 and 90. The test is frequently used in Dutch and Flemish education and its psychometric properties have been established in a sample of 10,059 Flemish children (Ghesquière and Ruijssenaars 1994). In previous analyses in the project sample, the 4-month test–retest reliability ranged between ρ 0.84 and 0.87 (Van de Weijer-Bergsma et al. 2015). The total number of problems answered correctly showed a moderate-to-strong correlation with performance on the math-problem-solving test, after controlling for grade (partial ρ between 0.50 and 0.51). In grade 2, students only finished the first two sets (addition and subtraction), while in higher grades, students finished all sets. The total number of problems answered correctly was scored.

### 2.4. Procedure

The measurements for the data used in this study took place at two occasions during the school year of 2012–2013, in September–October 2012 (T1) and January–February 2013 (T2). At T1, visual–spatial WM was assessed using the Lion game. Teachers received an email containing login information for their class of children and were asked to let all students finish the task within a period of three weeks. Math emotions were group-assessed with a paper-and-pencil questionnaire under the supervision of a research assistant. In grades 2 and 3, the research assistant read each question aloud, after which the students wrote down their answer. In grades 4 to 6, students completed the questionnaire independently after receiving instructions. At T2, we assessed verbal WM using the Monkey game, using a similar procedure to the Lion game. The arithmetic test was also administered by the teacher at T2. Children in grade 2 finished only the first two columns since multiplication is only introduced later during the school year of grade 2 and division is introduced in grade 3. The Cito Math test was administered by the classroom teacher at T2 as part of the standard national achievement testing procedure.

### 2.5. Missing Data

Data were available for a total number of 4471 children. Data could be missing at the item (e.g., one missing item for a subscale) or unit level (e.g., variable is completely missing). There were missing data at the item level for enjoyment (1 item: 62 children; 2 items: 3 children) and math anxiety (1 item = 47 children; 2 items = 5 children; 3 items = 1 child). Children with more than 1 item missing were excluded from the analysis. For children with 1 item missing, a mean score was calculated based on the available items.

There were also missing data at the unit level for *n* = 963 (21%) on the Monkey game, and for *n* = 637 (14%) on the Lion game. For enjoyment and math anxiety, data were missing for *n* = 432 (10%) and *n* = 418 (9%) children, respectively. For the Cito Math test, data were missing for *n* = 674 (15%) children. For the speeded arithmetic test, there were missing data for *n* = 474 (11%) children.

We only included cases with complete data in the analyses. In total, *n* = 2985 (67%) children had complete data on the variables included in the Cito Math test analysis. For this analysis, there were complete data for *n* = 633, 556, 602, 598, and 596 children in grades 1, 2, 3, 4, 5, and 6, respectively. For the analysis with the speeded arithmetic test, *n* = 2909 (61%) children had complete data. For this analysis, there were complete data for *n* = 570, 536, 611, 603, and 589 children in grades 1, 2, 3, 4, 5 and 6, respectively.

The large scale of the study made it unfeasible to keep track of reasons for missingness. However, several reasons are identified as highly probable. Missing data for many variables (at the unit level) were most probably due to absence from school during the time of testing (e.g., due to sickness or dentist visits). In a few cases, a technical error disturbed the data collection with the Monkey and Lion game. For the Cito Math test, teachers sometimes did not provide test results for a few students, and it is unknown if the test was administered later. In some cases, teachers did not provide test results for any students in their classrooms, suggesting that they did not administer the test at all, presumably because it was not part of the school policy.

### 2.6. Data Preparation and Analyses

Scores on math anxiety, math enjoyment, verbal WM, and visuospatial WM were z-transformed for the analyses. The scores for Verbal WM and Visuospatial WM were averaged to create a WM score. For this WM score, and for speeded arithmetic and math problem solving, grade-residualized scores were created by running one-way ANOVAs with grade as a factor and taking the standardized residuals. To create a standardized composite WM variable, the mean of the standardized residuals for the two WM tasks was taken and this score was z-transformed once more.

Mixed effects models were run using the lme4 package. In the first step, math performance was predicted with the predictors of WM, anxiety (both linear and quadratic), enjoyment (both linear and quadratic), and the interaction between WM on the one hand and the anxiety and enjoyment terms on the other hand. In the second step, interaction terms with grade were added to each predictor to investigate possible developmental effects. In the case of a significant interaction with grade, the model in step 1 was run for each grade separately in order to interpret the interaction effect. This procedure was carried out twice for speeded arithmetic and math problem solving separately. *p*-values of the effects were determined with conditional F-tests using Kenward–Roger degrees of freedom, using the afex package (Singmann et al. 2021).

More details, e.g., how we planned to deal with nonconvergence, violations of assumptions, outliers, and influential cases, can be found in the study preregistration (https://osf.io/wx7dt).

## 3. Results

### 3.1. Descriptive Statistics

The descriptive statistics by grade are displayed in Table 1. A correlation matrix of the interrelations in the entire sample (using the grade-residualized math and WM scores) is shown in Table 2. All interrelations were significant, which is also a reflection of the large sample size. As expected, math was positively related to WM ability and enjoyment, and negatively related to anxiety.

### 3.2. Math Problem Solving

First, the general model with emotions (linear and quadratic effects), WM, and their interaction was run for math problem solving. The results are shown in Table 3, Model 1.

Model 1 shows that WM and enjoyment were significant positive predictors of math performance, while anxiety had a negative main effect. The quadratic effects or interactions between WM and the other predictors were not significant.

In Model 2, interaction terms with grade were added to each predictor of Model 1. The same main effects were significant as in Model 1 and, furthermore, one interaction effect was significant: the effect of anxiety x grade. The negative effect of this interaction term shows that the negative effect of anxiety on performance was stronger in the higher grades. Follow-up analyses in which the model was run separately by grade showed a pattern of an effect of anxiety that increased in strength in grades 2–4, after which the effect stabilized (grade 2: b = −0.14, *p* = .001; grade 3: b = −0.20, *p* < .001; grade 4: b = −0.28, *p* < .001; grade 5: b = −0.24, *p* < .001; and grade 6: b = −0.28, *p* < .001).

### 3.3. Speeded Arithmetic

The same mixed effects models were run with speeded arithmetic as the dependent variable. First, the general model with emotions (linear and quadratic effects), WM, and their interaction effects was run. The results are shown in Table 4, Model 3. The results are largely similar to the results of math problem solving. As in the previous analysis, there were significant positive main effects of WM and enjoyment and a significant negative main effect of anxiety. In addition, there was also a significant negative interaction effect of WM x anxiety. This effect shows that the negative effect of anxiety was stronger for those with higher WM ability.

Again, in the next step, interaction effects with grade were added. The results are shown in Model 4. The results show the same effects as in Model 3 and also a significant negative interaction of anxiety x grade, as was also found for the math-problem-solving test. The negative effect of anxiety was, thus, the strongest in the higher grades. Follow-up analyses in which the model was run separately by grade showed a pattern of an effect of anxiety that was not significant in grade 2 and stable in the higher grades, with a small peak in grade 5 (grade 2: b = −0.07, *p* = .253; grade 3: b = −0.17, *p* < .009; grade 4: b = −0.18, *p* = .002; grade 5: b = −0.24, *p* < .001; and grade 6: b = −0.18, *p* = .004).

## 4. Discussion

In this study, we investigated the interplay of cognitive and affective factors in predicting mathematics achievement in primary school. Furthermore, potential interactions with grade level and differences between two types of math test were investigated. This large-scale study adds to the existing literature by applying the same analyses on a large data set spanning the larger part of primary school, and by including measures of both positive and negative emotions, in addition to WM. The inclusion of not only linear but also quadratic effects adds to the completeness of the study.

### 4.1. Main Effects of WM and Emotions

As hypothesized based on the previous literature (e.g., Barroso et al. 2021; Friso-Van den Bos et al. 2013; Namkung et al. 2019; Pinxten et al. 2014; Putwain et al. 2018), we found main effects of WM and emotions on mathematics achievement. As expected, students with stronger WM abilities and higher enjoyment performed better, whereas students with higher anxiety performed worse. Although this was true for both math problem solving and speeded arithmetic, the strength of the relations differed somewhat between the two types of tests: WM and anxiety were stronger predictors for math problem solving than for speeded arithmetic. This is partially in line with previous studies showing that the effect of mathematics anxiety is stronger for tasks requiring more complex math problem solving, and high-stakes tests (Namkung et al. 2019). Apparently, these aspects of the math-problem-solving test in our study weighed stronger than the speeded nature of the arithmetic task. The predictive value of enjoyment, on the other hand, was stronger for speeded arithmetic. The results suggest that enjoyment is more important for a low-stakes test: when there are no extrinsic motivators to exert effort in a test, intrinsic motivation or enjoyment becomes more important, which is also in line with previous research (Sundre and Kitsantas 2004).

Furthermore, on neither test did we find curvilinear effects on performance: neither for anxiety nor for enjoyment. A possible explanation, which is also a limitation of the current study, is that enjoyment and anxiety for mathematics were measured with a questionnaire about enjoyment and anxiety for mathematics in general. Unlike other questionnaires, such as the Mathematics Anxiety Rating Scale (Suinn and Winston 2003), the current questionnaire did not make a distinction between various achievement contexts such as tests, mathematics lessons, and homework. The idea that achievement emotions may depend on the specific situation (e.g., the type of test) has recently received more attention and has led Eccles and Wigfield (2020) to expand their expectancy–value theory to a situated expectancy–value theory. Possibly, more fine-grained, situated measures that are more directly related to a testing situation in mathematics would be necessary to detect a potential optimal level of anxiety or enjoyment. For example, an interesting avenue for future research might be complementing traditional questionnaires with physiological measures such as heart rate or electrodermal activity that are more direct measures of physiological arousal, while the students work on mathematics (Horvers et al. 2021). This is, however, a complex relation: one such study in undergraduate students showed that a relation between math anxiety questionnaire scores and electrodermal activity was only present for those who perceived low situational control while valuing the math activity as high—in other words, during relatively stressful circumstances (Strohmaier et al. 2020).

A second explanation is that anxiety was overall low in our sample, corroborating a cross-cultural study in Europe and Asia in which math anxiety in the Netherlands was the lowest of all participating countries (Morony et al. 2013), which may have led to a restriction of range that makes it more difficult to detect (curvilinear) relations.

### 4.2. Interactions between WM and Emotions

Another main goal of this study was to investigate whether and how WM interacts with anxiety and enjoyment in predicting mathematics achievement. For anxiety, several previous studies have indicated that students with a high WM ability suffer more from anxiety than students with a lower WM ability (Beilock and Carr 2005; Ramirez et al. 2013, 2016; Vukovic et al. 2013). This finding was partially replicated in the current study: we did find this interaction between anxiety and WM, but only on the speeded arithmetic test. This is an interesting finding, especially since the main effects of WM and anxiety on math performance were stronger for the other math test, the math-problem-solving test. However, in the math-problem-solving test, students do have the opportunity to reduce WM load by taking notes. They also have sufficient time to think over their answer carefully. The speeded arithmetic test may be less anxiety-provoking on average, but may, nevertheless, hamper students with stronger WM ability more due to its speeded nature. One explanation might be that it is more difficult to retrieve math facts from long-term memory under time pressure. A second explanation for the discrepancy lies in the nature of the test. Besides the speeded nature, the test starts with easy single-digit problems and then increases in difficulty to double-digit problems. The latter type requires more WM resources from the more advanced children that make it that far within the time limit. Those children, therefore, also have the largest potential to be affected by anxiety, if present.

We also explored the interaction effects between enjoyment and WM. We did not find such an effect, thus finding support for neither of the two competing hypotheses. Enjoyment was not more important for children with low WM ability, to use their limited resources effectively, and nor did it enable children with high WM ability the most to employ more complex strategies in a positive broaden-and-build mood. With the large sample in our study, we had over 99% power to detect even a small-sized effect of f2 = 0.01, so our null finding is not likely the result of a lack of statistical power. One explanation could be that the broaden-and-build mood, which is supposed to arise from enjoyment, might be more relevant for tasks that require more creativity in mathematical problem solving (e.g., Lin and Cho 2011) compared to the tests that we used, which rely more on the application of standard mathematical procedures. Secondly, as we argued before, our methodology may not have been sufficiently fine-grained or situated to detect interaction effects between enjoyment and working memory. Situated measures of math emotions could be combined WM assessments and more experimental manipulations of WM, for example with a dual-task paradigm, as previously applied by Ashcraft and Krause (2007).

### 4.3. The Effects of Grade Level

We also investigated if the relations between emotions and cognition were stronger at higher grade levels. We expected this to be the case, because older children had had more time for accumulating the effects of both anxiety and enjoyment, and better-developed self-awareness and metacognitive skills. The descriptive statistics (see Table 1) confirm that children in higher grades had higher levels of WM and anxiety and lower levels of enjoyment. However, grade level only interacted with anxiety, indicating that only the effect of anxiety was different across grades. Anxiety was a stronger predictor for math achievement in higher grade levels, and this was the case for both types of mathematics tests (problem solving and speeded arithmetic). Thus, math achievement seems to be more negatively affected by anxiety as students grow older. Notably, the interaction effect between anxiety and grade level on math achievement was strongest for the math-problem-solving test. An explanation might be that this test is repeatedly administered over the school years and students receive normative feedback on this test. As a result, the relation between anxiety and achievement may grow stronger over time for the math-problem-solving test as students become more aware of its high-stakes nature. In contrast, the content of the speeded arithmetic test was the same for all grade levels (although students in higher grades may reach the more complex calculations within the given time) and this test is not part of schools’ regular testing policy.

The finding that there were no three-way interactions between grade, WM, and emotions indicates that the age-related changes in the predictive value of anxiety for math achievement are similar for students with lower as well as higher WM.

### 4.4. Implications

This study has several potential implications. First, although our study examined general math emotions instead of situated math emotions, our findings from the two different math tasks suggest that situational factors such as the types of questions or the use of a speed limit may play an important role. Our findings suggest that these situational factors are also relevant for intervention development. Interventions aimed at preventing math anxiety are often focused on increasing students’ competences or removing worries (e.g., by teaching students relaxation techniques or different cognitive appraisals), or the type of feedback that teachers give their students (Ramirez et al. 2018). Besides interventions that solely focus on the students and the teachers, it might be desirable to reconsider testing policies (at the school or societal level) that potentially affect math anxiety and the type of feedback teachers give.

Second, teachers could be made aware that mathematics anxiety is already a relevant factor at a young age: already in grade 2, when children are around 7. They could also be made aware that this negative relation between mathematics anxiety and achievement increases with age. While teachers might presume that this is mainly an issue for students with low mathematics achievement or low WM skills, our findings indicate that students with high WM skills may be even more negatively affected by mathematics anxiety. More research is necessary to unravel the processes by which anxiety interacts with WM, and how this interacts with the type of mathematics test. In the long run, this might provide indications on how students can be helped to reduce the negative effects of anxiety. However, it should be stressed that the current findings do not provide direct evidence that anxiety causes lower mathematics achievement or that reducing anxiety would necessarily have a positive effect on students’ subsequent mathematics achievement.

Finally, practical implications regarding enjoyment are hard to identify based on the current study. More research in this area is needed to gain a better understanding of the cognitive processes that may come into play when a student experiences enjoyment while working on a mathematics task.

## 5. Conclusions

This study confirms and extends the findings of previous studies by demonstrating that both cognitive and affective factors play a role in predicting students’ mathematics achievement in primary school. The current study confirms previous evidence that lower WM ability, higher math anxiety, and lower enjoyment predict lower math performance, and that anxiety attenuates the positive effect of WM. It extends previous knowledge by showing that these effects were stable from grade 2 to grade 6, with the exception of the effect of anxiety, which was stronger in higher grades. It also extends previous research by showing that enjoyment did not affect the strength of the relation between WM and performance in the way that anxiety did, and by showing that effects of anxiety and enjoyment were not curvilinear.

Finally, the study revealed some task-specific effects. While WM and math anxiety were stronger predictors for math problem solving, math enjoyment was a stronger predictor for speeded arithmetic. Interestingly, however, math anxiety especially seemed to disturb higher-WM-ability students’ performance when performing speeded arithmetic, but not when working on math-problem-solving tasks.

## Figures and Tables

**Table 1:** Descriptive statistics by grade.

	Grade 2	Grade 3	Grade 4	Grade 5	Grade 6
	M(SD)	M(SD)	M(SD)	M(SD)	M(SD)
Enjoyment	2.90(0.96)	2.85(0.98)	2.83(0.90)	2.67(0.84)	2.59(0.81)
Anxiety	1.53(0.65)	1.61(0.71)	1.70(0.65)	1.67(0.65)	1.75(0.63)
WM ^1^	−0.68(0.82)	−0.23(0.78)	0.12(0.71)	0.29(0.69)	0.51(0.65)
Math problem solving ^2^	54.12(14.99)	74.33(14.91)	87.25(13.10)	101.33(12.33)	111.80(12.40)
Speeded arithmetic ^2^	30.58(8.67)	37.55(9.51)	44.78(9.07)	50.58(9.43)	55.01(9.15)

^1^ WM = working memory, standardized composite measure, before grade residualization (*M* = 0 in entire sample, across grades). ^2^ Scores prior to grade residualization.

**Table 2:** Correlations between the study variables.

	Math ^1^	Anxiety	Enjoyment	WM ^2^
Math ^1^		−0.24 ***	0.25 ***	0.27 ***
Anxiety	−0.31 ***		−0.25 ***	−0.13 ***
Enjoyment	0.20 ***	−0.26 ***		0.09 ***
WM ^2^	0.43 ***	−0.13 ***	0.08 ***	

^1^ Below diagonal: math problem solving. *n* = 2985. Above diagonal: speeded arithmetic. *n* = 2905. ^2^ WM = working memory. *** *p* < .001.

**Table 3:** Results of the mixed effects models with math problem solving as the dependent variable.

	Model 1					Model 2				
	*B*	*SE*	*df*	*F*	*p*	*B*	*SE*	*df*	*F*	*p*
Intercept	−0.01	0.35	1, 148.49	0.21	.648	−0.01	0.03	1, 152.70	0.16	.689
**Main effects**										
Grade						0.03	0.02	1, 176.68	3.06	.082
WM	**0.38**	**0.02**	**1, 130.26**	**367.03**	**<.001**	**0.38**	**0.02**	**1, 152.73**	**333.54**	**<.001**
Anxiety	**−0.23**	**0.02**	**1, 118.38**	**141.53**	**<.001**	**−0.23**	**0.02**	**1, 112.79**	**158.37**	**<.001**
Anxiety ^2^	0.03	0.02	1, 100.13	1.94	.166	0.02	0.02	1, 101.30	1.57	.213
Enjoyment	**0.12**	**0.02**	**1, 135.02**	**38.25**	**<.001**	**0.12**	**0.02**	**1, 139.51**	**41.90**	**<.001**
Enjoyment ^2^	0.01	0.02	1, 132.89	0.15	.702	0.00	0.02	1, 134.32	0.04	.840
**Interaction effects with WM**										
WM × anxiety	−0.04	−0.02	1, 86.66	2.98	.088	−0.03	0.02	1, 91.51	2.48	.119
WM × anxiety ^2^	−0.00	0.02	1, 86.64	0.02	.878	0.01	0.02	1, 89.45	0.08	.776
WM × enjoyment	0.01	0.02	1, 102.31	0.28	.598	0.01	0.02	1, 125.10	0.15	.701
WM × enjoyment ^2^	0.00	0.02	1, 113.11	0.00	.990	0.00	0.02	1, 121.37	0.02	.891
**Interaction effects with grade**										
WM × grade						−0.01	0.01	1, 156.81	1.07	.302
Anxiety × grade						**−0.03**	**0.01**	**1, 126.34**	**5.61**	**.019**
Anxiety ^2^ × grade						−0.02	0.01	1, 112.59	1.83	.179
Enjoyment × grade						0.01	0.01	1, 152.43	0.29	.589
Enjoyment ^2^ × grade						0.00	0.01	1, 147.36	0.02	.892
WM × anxiety × grade						0.01	0.01	1, 107.16	0.33	.567
WM × anxiety ^2^ × grade						0.02	0.01	1, 90.16	1.15	.286
WM × enjoyment × grade						0.02	0.01	1, 127.88	1.39	.240
WM × enjoyment ^2^ × grade						0.00	0.01	1, 134.69	0.01	.903
Nakagawa’s marginal *R*^2^				.286					.290	

Notes: WM = working memory; ^2^ denotes a quadratic term. Terms in boldface are significant (*p* < .05).

**Table 4:** Results of the mixed effects models with speeded arithmetic as the dependent variable.

	Model 3					Model 4				
	*B*	*SE*	*df*	*F*	*p*	*B*	*SE*	*df*	*F*	*p*
Intercept	0.00	0.03	1, 144.01	0.03	.873	0.00	0.03	1, 147.46	0.00	.949
**Main effects**										
Grade						0.01		1, 175.10	0.30	.583
WM	**0.22**	**0.02**	**1, 115.25**	**135.31**	**<.001**	**0.23**	**0.02**	**1, 139.73**	**125.76**	**<.001**
Anxiety	**−0.17**	**0.02**	**1, 111.66**	**76.43**	**<.001**	**−0.17**	**0.02**	**1, 109.17**	**75.98**	**<.001**
Anxiety^2^	0.03	0.02	1, 94.86	1.70	.195	0.03	0.02	1, 92.98	1.66	.201
Enjoyment	**0.20**	**0.02**	**1, 128.82**	**104.46**	**<.001**	**0.21**	**0.02**	**1, 134.04**	**106.73**	**<.001**
Enjoyment^2^	**−**0.03	0.02	1, 129.15	2.10	.150	**−**0.03	0.02	1, 129.37	1.79	.184
**Interaction effects with WM**										
WM × anxiety	**−0.05**	**0.02**	**1, 86.87**	**4.71**	**.033**	**−0.05**	**0.02**	**1, 87.94**	**5.06**	**.027**
WM × anxiety ^2^	0.01	0.02	1, 79.66	0.37	.544	0.02	0.02	1, 73.92	0.57	.453
WM × enjoyment	**−**0.02	0.02	1, 97.56	1.50	.223	**−**0.02	0.02	1, 120.66	0.74	.390
WM × enjoyment ^2^	**−**0.02	0.02	1, 93.68	0.95	.333	**−**0.02	0.02	1, 97.80	0.69	.410
**Interaction effects with grade**										
WM × grade						0.01	0.01	1, 147.41	0.52	.473
Anxiety × grade						**–0.03**	**0.01**	**1, 121.62**	**4.86**	**.029**
Anxiety ^2^ × grade						0.00	0.01	1, 105.45	0.00	.967
Enjoyment × grade						0.00	0.01	1, 142.24	0.01	.913
Enjoyment ^2^ × grade						0.00	0.01	1, 142.01	0.00	.946
WM × anxiety × grade						**−**0.02	0.01	1, 102.93	1.42	.235
WM × anxiety ^2^ × grade						0.00	0.01	1, 74.83	0.00	.957
WM × enjoyment × grade						0.01	0.01	1, 121.10	0.37	.546
WM × enjoyment ^2^ × grade						**−**0.01	0.01	1, 111.94	0.74	.390
Nakagawa’s marginal *R*^2^				.178				0.180		

Note: WM = working memory; ^2^ denotes a quadratic term. Terms in boldface are significant (*p* < .05).

## Data Availability

Data available on request from the authors.
